# Environmental causes of between-population difference in growth rate of a high-altitude lizard

**DOI:** 10.1186/s12898-018-0194-8

**Published:** 2018-09-24

**Authors:** Hong-Liang Lu, Chun-Xia Xu, Zhi-Gao Zeng, Wei-Guo Du

**Affiliations:** 1 Key Laboratory of Hangzhou City for Ecosystem Protection and Restoration, School of Life and Environmental Sciences, Hangzhou Normal University, Hangzhou, 310036 Zhejiang China; 2 Key Laboratory of Animal Ecology and Conservation Biology, Institute of Zoology, Chinese Academy of Sciences, Beijing, 100101 China

**Keywords:** *Phrynocephalus vlangalii*, Elevational variation, Growth rate, Food availability, Activity period

## Abstract

**Background:**

Ectothermic animals living in cold (high latitude or high elevation) regions are predicted to grow slower due to limited thermal opportunities for activity and food resources than those living in warm regions. However, the Qinghai toad-headed lizards (*Phrynocephalus vlangalii*) grow faster and reach a larger adult size at a high-elevation site than at a low-elevation site. In this study, we aimed to identify the genetic and environmental causes of this between-population difference in growth rate by conducting mark-recapture and common garden experiments on juvenile growth rate, and investigating the thermal environment, lizard body temperature, potential prey availability at the two elevation sites.

**Results:**

Compared with low-elevation individuals, high-elevation juvenile lizards had higher growth rates in the field, but grew at similar rates in the laboratory. High-elevation lizards had higher active body temperatures than low-elevation lizards despite similar air temperatures in the period of field investigation. The high-elevation site had relatively more and larger preys than the low-elevation site.

**Conclusions:**

Inter-population difference in growth rate of *P. vlangalii* may primarily result from developmental plasticity in response to the difference in environmental resources, rather than genetic differentiation. The higher growth rate of high-elevation lizards is likely associated with higher potential food availability and higher active body temperatures.

## Background

Growth rate is a critical life history trait influencing individual fitness [1, 2]. For example, growth rate may determine the age and body size at maturity, survival or reproductive success [3–6]. Growth rate of organisms often vary along a geographic (e.g. latitudinal and altitudinal) gradient [7–9]. Such geographic variation in growth rate may not only stem from genotypic difference among populations, but also be attributable to environmental factors such as temperature and food availability, particularly in ectotherms [5, 10–14]. To better understand the role of growth rate in generating geographic variations in other life history traits, such as body size, we need to identify the relative roles of extrinsic versus intrinsic factors generating geographic variation in these traits [15, 16].

Altitudinal variation in growth rate has been reported in a number of species [9, 17–20]. However, the underlying mechanisms have received much less attention [but see 21–23]. Compared with the ectotherms living at low-elevation sites, the high-elevation ectotherms may grow slower due to the limited thermal opportunity for activity [6, 13, 24] or resource limitations [17, 25]. However, sometimes high-elevation ectotherms can grow faster than low-elevation ones due to greater food availability [21, 26, 27]. Those species in high-elevation (cold) regions may grow faster through the following ways that are not mutually exclusive. First, they may evolve fast-growing genotypes to compensate the disadvantage of a low growth rate due to cold environment [3, 15]. Second, environmental factors, including precipitation, food availability and sunshine duration, facilitate animals at colder sites to have higher growth rates than those at warmer sites [21]. Of those environmental factors that potentially induced growth rate variation, thermal regime and food availability are especially important because they may have strong proximate effects on the growth rate of animals [6, 14, 15, 24, 28–30]. High food availability and warm temperatures may enable ectotherms to grow fast [26, 31, 32]. In addition, most studies of altitudinal variation in reptile life history are within the altitudinal range of 150–2000 m [6, 13, 14, 21, 24, 27, 33–35], with few studies carried out in the regions above 2000 m in elevation [26, 36–38]. With an average elevation exceeding 4500 m, the Qinghai-Tibetan plateau region offers an opportunity for studying altitudinal variation in life history traits of organisms.

The Qinghai toad-headed lizard (*Phrynocephalus vlangalii*), which is widely distributed in the Qinghai-Tibetan Plateau with an elevation range from 2000 to 4500 m, provides an excellent model to study altitudinal variation of growth rate in reptiles. Our previous study indicated that *P. vlangalii* individuals at a high-elevation site (Maduo, 4250 m elevation) can grow faster and reach a larger adult size than those at a low-elevation site (Maqu, 2930 m elevation) [39]. In this study, we conducted a mark-recapture experiment in the field and a common-garden experiment in the laboratory to determine genetic *vs* environmental components of this between-population difference in juvenile growth rate. To identify the environmental causes of the between-population difference in growth rate, we further examined between-population differences in thermal environment, body temperature, diet composition, and potential food availability at these two sites separated by c.a. 1300 m elevation. We hypothesized that lizards from the high-elevation population would grow faster than those from the low-elevation population under identical laboratory conditions, if high-elevation lizards evolve fast-growing genotypes to compensate the disadvantage of harsh environment; or lizards from the high-elevation population would grow faster than those from the low-elevation population in the field rather than in the laboratory, if the between-population difference in lizard growth rate is induced primarily by environmental differences between the two sites (specifically, more food or thermal opportunities at the high-elevation site than at the low-elevation site).

## Methods

### Study species and study areas

*Phrynocephalus vlangalii* is a small ground-dwelling viviparous agamid lizard (up to 80 mm snout-vent length, SVL), and typically found in open spaces in arid or semi-arid regions covered by sparse vegetation in the Qinghai-Tibetan plateau. Many life-history traits of this species show large amounts of geographic variation [40–42]. Courtship occurs in May, and parturition occurs between mid-July and late August [43].

This study was conducted at a low-elevation site, Maqu (Gansu, western China, 34°00′N, 102°04′E, 2930 m elevation), and a high-elevation site, Maduo (Qinghai, western China, 34°55′N, 98°12′E, 4250 m elevation). Over a linear distance of 370 km, these two sites have distinct mean annual air temperatures (1.4 °C in Maqu vs − 1.7 °C in Maduo), rainfall (550 mm in Maqu vs 379 mm in Maduo), and sunshine duration (2594 h in Maqu vs 2842 h in Maduo). Toad-headed lizards are abundant in both study sites, and previous phylogenetic analysis has indicated that these two populations belong to a single lineage [44].

### Growth of juvenile lizards in the field and laboratory

In mid-June (10th‒18th) and late August (22nd‒30th) of 2011 and 2012, a study plot of 4000 m^2^ in each site (80 × 50 m^2^) was visited 4 times. At each visit, active lizards were captured by hand whenever possible, weighed and measured for SVL, noting sex and reproductive condition. In the years of 2011 and 2012, a total of 370 juveniles (low-elevation: 58 in 2011 and 75 individuals in 2012; high-elevation: 107 in 2011 and 130 individuals in 2012) were captured and marked individually by toe-clipping upon first capture. After collecting the data, lizards were released immediately at their site of capture. Data from the mark-recapture study were used to estimate the growth rate in the field.

In mid-July of 2011, some gravid females captured in the field (29 from the low-elevation and 30 from the high-elevation) were transferred to our laboratory in Hangzhou Normal University. Females gave birth from late July to mid-August in the laboratory. A total of 79 newborns (51 from the low-elevation and 28 from the high-elevation) were collected, and housed in fifteen 50 × 40 × 30 cm terraria (3–4 individuals per terrarium) that placed in a temperature-controlled room set at 20 ± 2 °C, with individuals from different populations being assigned evenly to each aquarium. A 60 W light bulb was suspended above each terrarium allowing thermoregulation for 10 h daily. Small mealworms (larvae of *Tenebrio molitor*), and water enriched with vitamins and minerals were provided ad libitum throughout the experiment. Newborns were weighed and measured for SVL at birth, and at an age of 30 days to determine postnatal growth in a month.

### Ambient temperature and lizard body temperature

From early June to late August of 2011, we placed 10 Thermocron iButton temperature loggers (DS1921, 711C, MAXIM Integrated Products/Dallas Semiconductor Ltd, Sunnyvale, USA) in each study site to record the ground surface temperature (*T*_g_) in the natural habitat at an interval of 3 h. Each iButton was pressed into in a silvery-white balloon and placed at a 0.5 cm depth from the surface. Additionally, during a 1-week period in early August 2011 (1st‒7th), the active lizards at both study sites were captured by hand, and their body (cloacal) temperatures (*T*_b_), *T*_g_ and air temperatures at 10 cm above the substrate (*T*_a_) were recorded immediately after capture using a UT325 electronic thermometer (Uni-trend Group, Shanghai, China) to investigate between-population difference in active body temperature of lizards.

### Diet composition and prey availability

In early August 2011 (1st‒7th), sixty-three adult lizards captured from the two study sites (15♀♀, 23 ♂♂ in the low-elevation population; 9♀♀, 16 ♂♂ in the high-elevation population) were immediately sacrificed for diet analysis. We dissected specimens, removed the stomachs, and placed the animal and its stomach in 10% formalin. Specimens were transferred to our laboratory in Hangzhou Normal University. Stomach contents were spread in a Petri dish and all prey items were identified to the taxonomic level of Order with the aid of a dissecting microscope in the laboratory.

Potential prey availability in the two sites was estimated by means of pitfall traps. Despite having a risk of biased estimation of prey availability, pitfall traps are considered as an effective technique to estimate ground-dwelling arthropods, which predominate in the diet composition of lizards. In the years of 2011 and 2012, we visited the study sites four times (early September 2011, mid-June, late July and late August 2012), and randomly placed thirty pitfalls (diameter = 20 cm, depth = 15 cm) throughout the study sites. All the trapped arthropods were collected after 48 h, and placed into wide-necked bottles with ethyl acetate and transferred to the laboratory for analysis. Arthropods captured in pitfall traps were identified to the taxonomic level of Order, counted and measured individually for body length and width. The volume (V) of each prey item was calculated using the equation of an ellipsoid: V = (4/3)πab^2^, where a = 1/2 body length and b = 1/2 body width. We used the total volume of prey as a measure of prey availability.

### Data analysis

We calculated size-specific and mass-specific growth rates during the experiments using the formula ln (measurement_2_/measurement_1_)/(date_2_ − date_1_). Prior to analysis, the normality of distributions and homogeneity of variances in the data were tested using the Kolmogorov–Smirnov test and Bartlett’s test, respectively. Analysis of variance (ANOVA) or covariance (ANCOVA) was used to determine between-population differences in body and ambient temperature, food availability and individual growth rate of lizards. Log-likelihood ratio test (*G*-test) was used to determine between-site difference in recapture rate and the proportion of the individuals having empty stomachs. Values are presented as mean ± standard error (SE), and the significance level is set at α = 0.05.

## Results

### Growth rate of juveniles in the field and laboratory

**Fig. 1:**
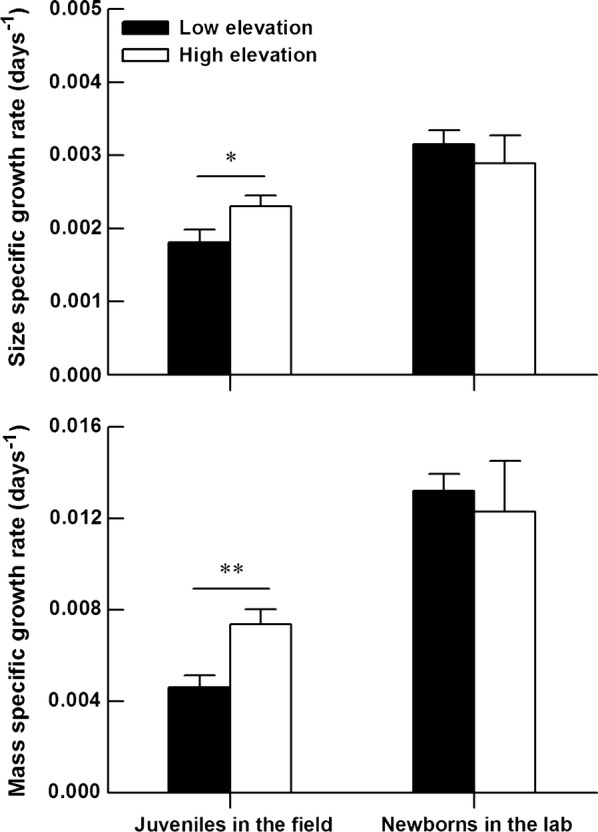
Mean values (+SE) of size- and mass-specific growth rates of juveniles in the field and newborns in the laboratory from two different elevation sites. The asterisks indicate significant differences (*P < 0.05, **P < 0.01)

A total of 81 juveniles (30 from the low-elevation population and 51 from the high-elevation population) were recaptured at least once during the mark-recapture experiment. The proportion of recaptured individuals in total marked lizards did not differ between the two study sites (*G* = 0.50, *df* = 1, *P* = 0.817). Initial body size of recaptured lizards did not differ between sites (low-elevation vs high-elevation: SVL, 41.43 ± 0.73 vs 41.66 ± 0.39 mm, *F*_1, 79_ = 0.09, *P* = 0.762; mass, 2.78 ± 0.14 vs 2.80 ± 0.08 g, *F*_1, 79_ = 0.01, *P* = 0.934). Lizards at the high-elevation site increased their SVL (*F*_1, 79_ = 4.41, *P* = 0.039) and body mass (*F*_1, 74_ = 8.35, *P* < 0.01) more rapidly than those at the low-elevation site (Fig. 1).

Thirty-nine newborns (22 from the low-elevation population, 17 from the high-elevation population) died during the growth experiment. The proportion of survived individuals did not differ between the two study populations (*G* = 2.25, *df* = 1, *P* = 0.134). Initial body size of survived individuals from low-elevation population was greater than that from high-elevation population (mixed-model ANOVA with site of origin as a fixed factor and clutch as a random factor, low-elevation vs high-elevation: SVL, 28.8 ± 0.4 vs 26.7 ± 0.4 mm, *F*_1, 18.3_ = 6.03, *P* = 0.024; mass, 0.93 ± 0.05 vs 0.73 ± 0.05 g, *F*_1, 19.1_ = 3.15, *P* = 0.092). In the first month, the newborns did not differ in either size-specific growth rate (*F*_1, 8.6_ = 0.81, *P* = 0.394), or mass-specific growth rate (*F*_1, 14.3_ = 0.22, *P* = 0.645) between populations (Fig. 1).

### Ambient temperature and lizard body temperature

**Fig. 2:**
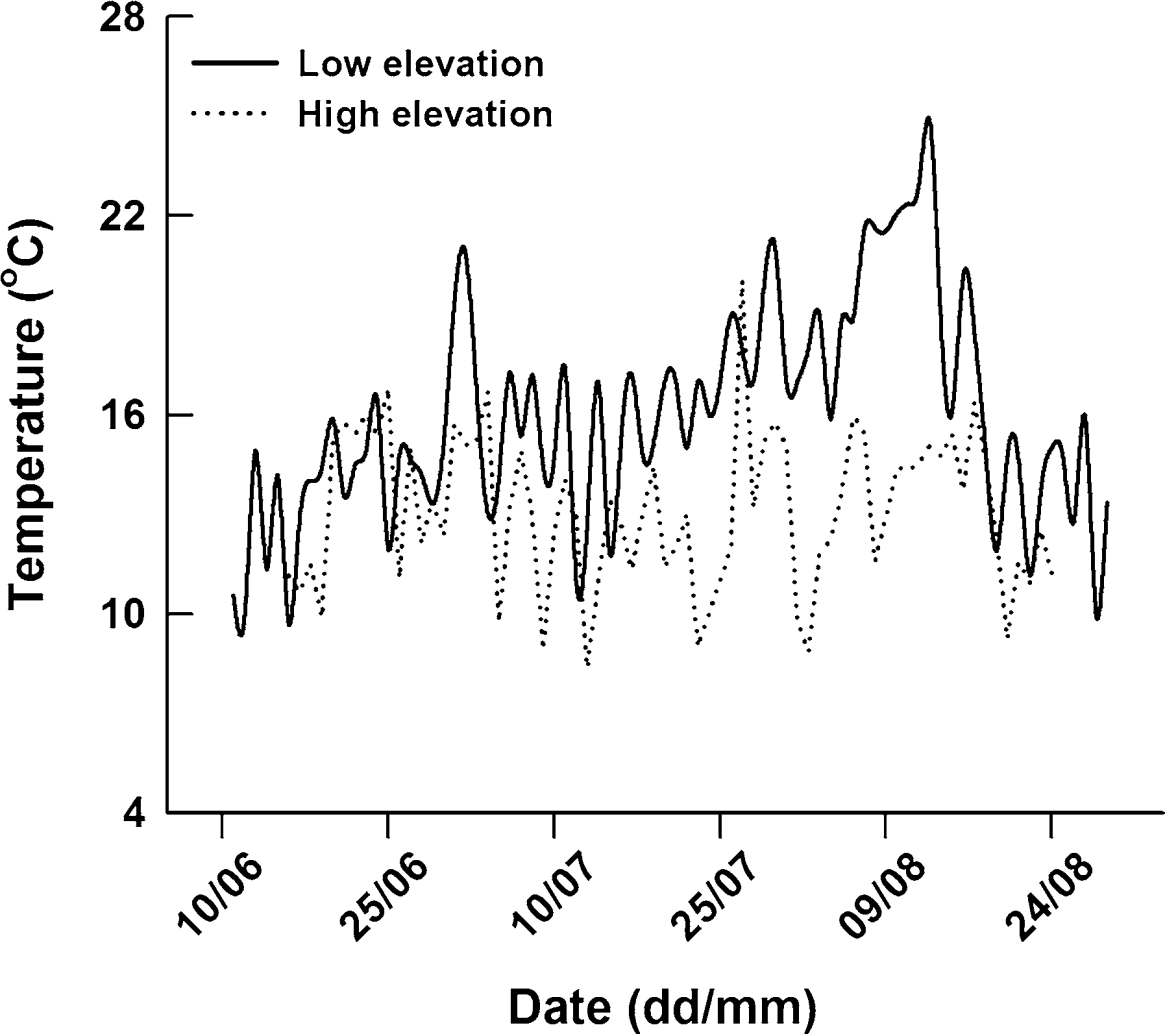
Ground surface temperatures (from early June to late August) in natural habitats used by *Phrynocephalus vlangalii* at two different elevation sites

**Fig. 3:**
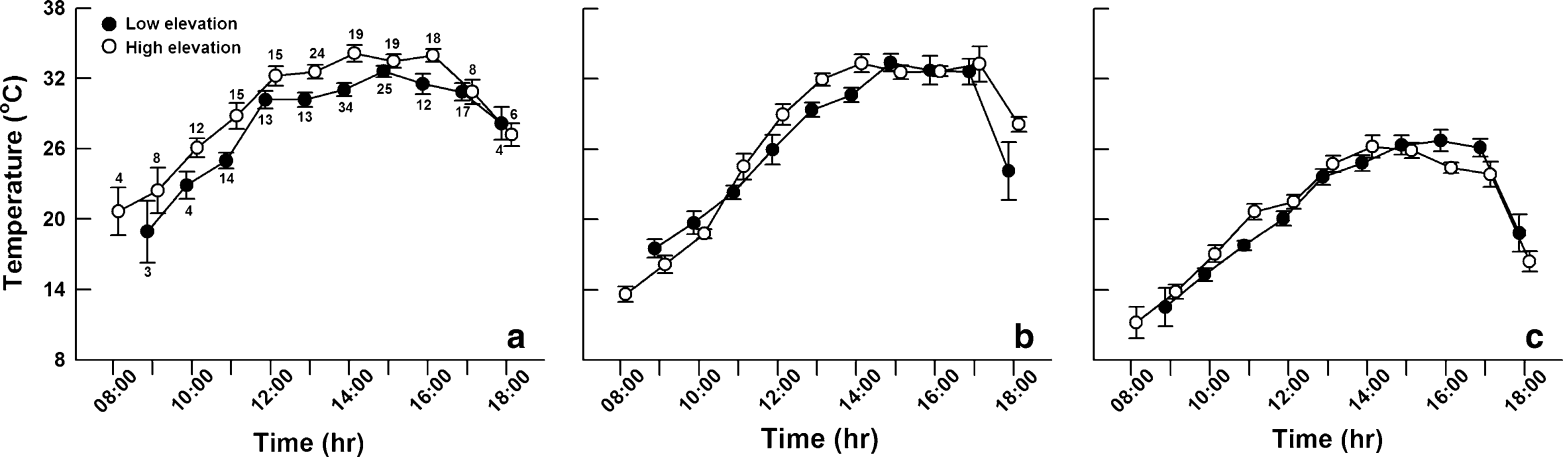
Body temperatures of *Phrynocephalus vlangalii* (**a**), ground surface temperatures (**b**) and air temperatures at 10 cm above the substrate (**c**) during a 1-week period in August at two different elevation sites. Numbers above (or below) the error bars represent sample sizes

The records from iButton temperature loggers showed that the mean daily ground surface temperatures (*T*_g_) in the low-elevation and high-elevation site were 16.0, and 13.1°C, with ranges of 9.7–24.8 and 8.4–20.0 °C, respectively (Fig. 2). During the period of field investigation (from August 1st to 7th in 2011), lizard body temperatures (*T*_b_) were significantly higher than air temperatures (*T*_a_) at both low- and high-elevation sites (paired *t*-test: low-elevation, *t* = 20.65, *df* = 138, *P* < 0.001; high-elevation, *t* = 27.85, *df* = 147, *P* < 0.001). Lizard *T*_b_s were higher than *T*_g_s at high-elevation site (*t *= 7.58, *df* = 147, *P* < 0.001), but not at low-elevation site (*t* = 1.33, *df* = 138, *P* = 0.185) (Fig. 3). In the same time period, mean lizard *T*_b_s (*F*_1, 263_ = 21.57, *P* < 0.001) and *T*_g_s (*F*_1, 263_ = 8.05, *P* < 0.01) at high-elevation site were higher than those at low-elevation site, but mean *T*_a_s did not differ between the two sites (*F*_1, 263_ = 0.06, *P* = 0.802) (Fig. 3). Lizard *T*_b_s were positively correlated with *T*_g_s in both populations (low-elevation, *r*^2^ = 0.48, *F*_1, 137_ = 124.15, *P* < 0.001, *T*_b_ = 14.87 + 0.52*T*_g_; high-elevation, *r*^2^ = 0.63, *F*_1, 146_ = 244.03, *P* < 0.001, *T*_b_ = 14.28 + 0.58*T*_g_). Between-site difference in *T*_b_ was still evident after removing the effect of *T*_g_ (ANCOVA with *T*_g_ as the covariate, *F*_1, 262_ = 14.02, *P* < 0.001).

### Diet composition and prey availability

Five lizards from the low-elevation population and one from the high-elevation population had no prey items in their stomachs. The proportion of individuals having empty stomachs did not differ significantly between the high-elevation (4.0%) and the low-elevation (13.2%) populations (*G* = 1.64, *df *= 1, *P* > 0.10). The wet masses of stomach contents were greater in high-elevation lizards (0.21 ± 0.02 g, *n* = 24) than those in low-elevation ones (0.15 ± 0.02 g, *n* = 33) (ANCOVA with body mass as the covariate, *F*_1, 54_ = 4.56, *P* = 0.037). The diet of *P. vlangalii* was primarily made up of arthropods. Coleoptera, Diptera, Lepidoptera and Hymenoptera (ants) were recorded from specimens in both populations. Lithobiomorpha and Opiliones were only recorded from specimens collected at the high-elevation site, while plant material was only found at the low-elevation site. Three of six major taxa (Coleoptera, Diptera and Lepidoptera) accounted for approximately 80% of the total number, and 90% of the volumetric contribution of food ingested by the lizards from both study sites (Table 1).

**Table 1:** Prey items found in stomach contents of adult *Phrynocephalus vlangalii* collected from two different elevation sites

Prey type	Low-elevation site			High-elevation site		
	Prey number	Proportion of total prey number (%)	Proportion of total food volume (%)	Prey number	Proportion of total prey number (%)	Proportion of total food volume (%)
Coleoptera	164	55.4	59.1	115	52.0	33.0
Diptera	34	11.5	13.3	67	30.3	33.1
Lepidoptera	31	10.4	16.3	25	11.3	24.9
Hymenoptera	63	21.3	3.4	11	5.0	5.9
Lithobiomorpha	–	–	–	2	0.9	2.7
Opiliones	–	–	–	1	0.5	0.4
Plant material	4	1.4	7.9	–	–	–

**Fig. 4:**
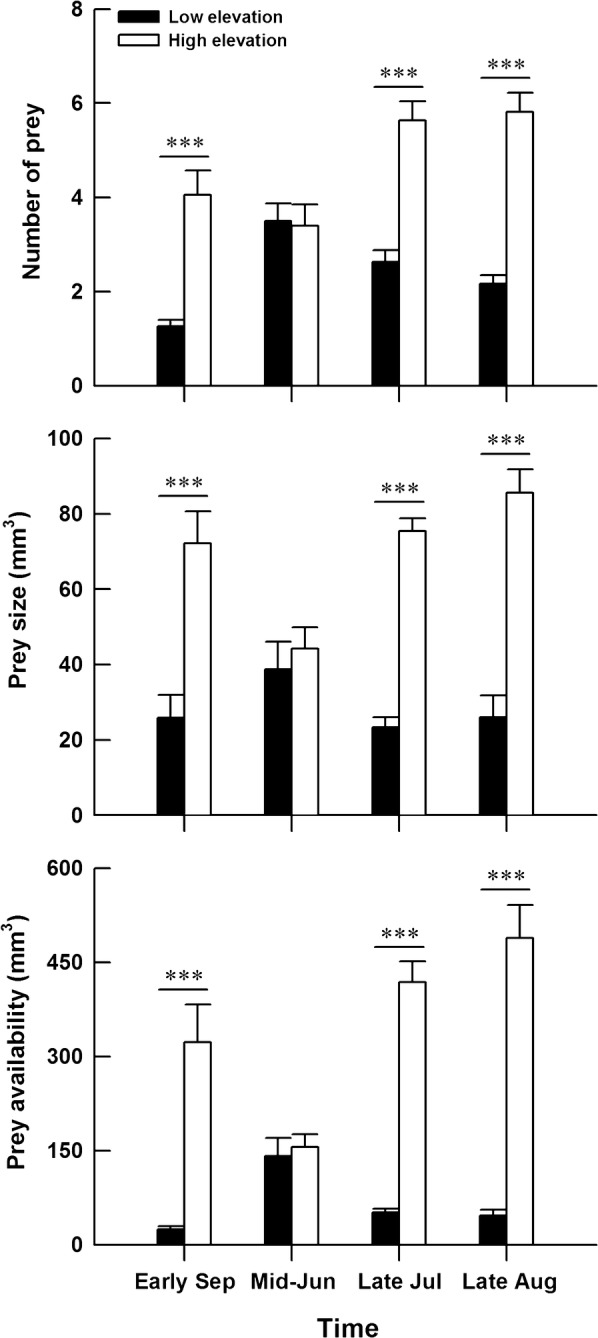
Mean values (+SE) of potential prey size and availability in natural habitats used by *Phrynocephalus vlangalii* at two different elevation sites. The asterisks indicate significant differences (***P < 0.001)

The two study sites possess similar types of prey (Table 2). However, the proportion of some prey differed between the two sites. The high-elevation site had higher proportions of Lepidoptera and Opiliones, whereas the low-elevation site had higher proportions of Hymenoptera and Diptera (Table 2). The availability and size of the three major taxa (Coleoptera, Diptera and Lepidoptera) of prey for the lizard differed significantly between the two sites, with more and larger prey at the high-elevation site than at the low-elevation site (Table 3, Fig. 4).

**Table 2:** Potential prey items from pitfall traps placed in natural habitats used by *Phrynocephalus vlangalii* at two different elevation sites

Prey type	Low-elevation site		High-elevation site	
	Prey number	Proportion of total prey number (%)	Prey number	Proportion of total prey number (%)
Coleoptera	86	42.0	312	52.7
Hymenoptera	53	25.9	1	0.2
Diptera	49	23.9	26	4.4
Lepidoptera	1	0.5	41	6.9
Opiliones	4	1.9	201	33.9
Lithobiomorpha	12	5.8	11	1.9

**Table 3:** Results of repeated measures ANOVA (with site of origin as the between-subject factor and season as the within-subject factor) for the availability of potential prey in natural habitats used by *Phrynocephalus vlangalii* at two different elevation sites

	Number of prey	Prey size	Prey availability
Site of origin	*F*_1, 58_ = 89.21, *P* < 0.001	*F*_1, 58_ = 125.50, *P* < 0.001	*F*_1, 58_ = 77.98, *P* < 0.001
Season	*F*_3, 174_ = 6.59, *P* < 0.001	*F*_3, 174_ = 5.65, *P *< 0.01	*F*_3, 174_ = 2.02, *P* = 0.113
Site of origin × season	*F*_3, 174_ = 10.20, *P* < 0.001	*F*_3, 174_ = 16.44, *P* < 0.001	*F*_3, 174_ = 8.62, *P* < 0.001

## Discussion

The climate of the Qinghai-Tibetan Plateau is characterized by strong solar radiation, low temperatures and limited precipitation [45], which might affect the growth of those animal species inhabiting this area. Our study demonstrated significant variations in thermal environment, food availability and juvenile growth rate between low- and high-elevation populations of *P. vlangalii*. Under identical laboratory conditions, however, juvenile lizards from the two populations showed a similar growth rate. These results indicate that divergent growth rate of *P. vlangalii* is primarily derived from plasticity induced by environmental factors rather than genetic differentiation.

Different genotypes might be favored by natural selection in different environments [46, 47]. Fast growing genotypes are often found in those resource-poor environments causing slow growth [7]. For example, temperate juveniles grew faster than tropical ones under identical laboratory conditions in the eastern water skink *Eulamprus quoyii* [3]. Similarly, genetic differences in growth rate have been documented using the common garden or reciprocal transplant experiment in other reptile populations [2, 14, 48, 49]. However, our laboratory growth experiment revealed that newborns of *P. vlangalii* from the two study populations had similar growth rates, which was inconsistent with our first prediction. Similarly, no significant genetic differentiation in growth rate was also reported in the common lizard, *Zootoca vivipara* [6].

**Fig. 5:**
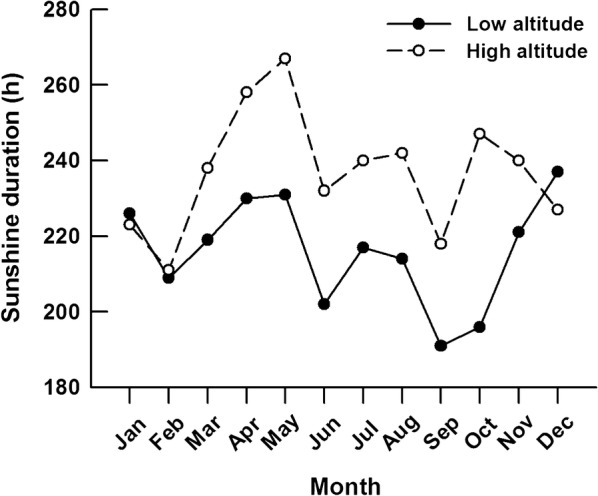
Monthly mean sunshine duration for the two study sites where *Phrynocephalus vlangalii* were collected (data from http://data.cma.cn/)

Temperature and food quality are likely to be the most important environmental factors influencing lizard growth rates [13, 21]. Due to thermal constraints on body temperatures, lizard growth should be positively correlated with the amount of time over which body temperatures are suitable for activity. An increase in thermal opportunity for activity can increase growth rate of lizards [14, 26]. Due to relatively higher ambient temperatures, the low-elevation regions presumably offer longer potential daily and seasonal activity periods, and thereby tend to produce greater growth rates and prolonged growth periods [26]. However, *P. vlangalii* individuals at the low-elevation site did not necessarily have higher body temperatures than those at the high-elevation site (Fig. 3). In fact, our survey results in August showed that, in the same time period, *T*_a_s at the low-elevation site were not higher, but *T*_b_s and *T*_g_s were lower than at the high-elevation site. Unfortunately, we lacked the data on operative temperature here, so the *T*_g_s recorded by iButton temperature loggers [> minimal recorded value of *T*_g_ (12 °C) when lizards were active] were used to estimate lizard *T*_b_s over the active season. There was also no significant between-population difference in estimated *T*_b_ (*t* = 0.46, *df* = 414, *P* = 0.647). Solar radiation increases with increasing elevation [50]. Hence the higher intensity of solar radiation at the high-elevation site contributes to higher ground surface temperatures, and thus higher lizard body temperatures [51]. Moreover, high-elevation ectothermic animals can increase their active body temperatures in a more efficient way by using solar radiation heat sources, and/or by being active at low temperatures [52, 53]. For example, despite lower air temperature, high-elevation *Liolaemus* lizards had similar activity temperatures compared with low-elevation lizards [54]. Body size and coloration can affect thermoregulation efficiency of ectothermic animals [55, 56]. For example, some lizards living at high elevations have darker dorsal colorations to facilitate thermoregulation and warm faster than those at low elevations [55]. *Phrynocephalus* lizards in the Qinghai-Tibetan Plateau have an abdominal black-speckled area, which becomes larger with increased elevation. The black-speckled area allows lizards to gain heat more efficiently probably by absorbing reflected solar radiation from ground surfaces [57]. It would be of great interest to evaluate between-population differences in dorsal coloration and abdominal black-speckled area size and the contribution of the difference to thermoregulation efficiency of *P. vlangalii* in future studies. Overall, these findings together with a shorter annual sunshine duration at low-elevation site than at high-elevation site (Fig. 5, data from http://data.cma.cn/) allow us to infer that the daily and seasonal activity period of low-elevation lizards are not necessarily longer than that of high-elevation lizards. Therefore, *P. vlangalii* from the high-elevation population might be able to use thermal opportunities more efficiently to facilitate individual growth. Nonetheless, we only quantified lizard thermoregulation in a shorter period (1 week in summer), further extensive studies on the thermoregulation of lizards from the two populations are needed to test our hypothesis on altitudinal variation in thermal effects on lizard growth.

Consistent with our prediction, potential prey availability was more abundant at the high-elevation site than at the low-elevation site, and high-elevation juveniles grew faster than low-elevation ones. Actually, higher growth rates for high-elevation *P. vlangalii* were also confirmed in another study using field mark-recapture data and von Bertalanffy growth parameters [39]. As suggested by some previous studies, the effect of food availability on lizard growth rate is more immediate [27]. The observed inter-population difference in growth rate of *P. vlangalii* also results from the variability in habitat food availability. Interestingly, high-elevation newborns were smaller than low-elevation ones, but high-elevation adults were reported to have larger sizes than low-elevation ones [39–41]. These results imply that the environmental factors determining growth rate are large enough to override the variation in newborn size. The higher food resources at high-elevation site could allow *P. vlangalii* juveniles to grow faster and thus reach larger adult size. On the other hand, food scarcity may restrict the growth potential of juveniles at low-elevation site. Larger newborn sizes in this environment should be favored by natural selection to compensate for lower growth rates [58, 59].

Lizard growth rates can be regulated by complex interaction between those environmental factors such as thermal opportunity for activity and food availability [21]. Consequently, it may be difficult to predict the latitudinal and altitudinal patterns of growth rate in different species of lizards. For example, the canyon lizards (*Sceloporus merriami*) at an intermediate-elevation site grow fastest despite food availability increasing with elevation [24], while the common lizards (*Z. vivipara*) raised at a low-elevation site grow faster than those at the high-elevation site, resulting from between-site differences in thermal environments [6]. Additionally, other internal and external factors can have a significant impact on growth rates of lizards, such as the length of daily activity and costs of activity [13], and metabolic expenditure or activity constraints imposed by predation risk [26, 38]. Understanding the contribution of these factors to lizard growth would be an interesting topic for future research.

## Conclusions

Our study found that inter-population differences in growth rate of *P. vlangalii* were induced by the differences in environmental resources, rather than resulting from genetic differentiation. Although the slightly colder environments at the high-elevation site are thought to select for larger offspring that might have higher survival probability [35, 60, 61], smaller newborns were produced at the high-elevation site with more food resources. Fast growth, driven by high food availability, appeared to surpass the adverse effects of small body size and cold thermal environments. Contrarily, larger newborns produced at the low-elevation site ensured their survival, and compensated for slower growth rate in a low-food environment [21]. High-elevation lizards may not always have lower body temperatures and shorter activity period than low-elevation ones possibly due to more efficient ways to improve body temperature, higher solar radiation intensity and longer sunshine duration at the high-elevation site. Therefore, temperature might not be the major limiting factor that shapes altitudinal differences in growth rates. Our data indicated that potential food availability at high-elevation site allow juvenile *P. vlangalii* to have higher field growth rate and thereby larger body size at adulthood than at low-elevation site. Unfortunately, we only conducted a two-population comparison due to logistic difficulties in the current study. Further studies involving multiple populations along the altitudinal cline would be essential to elucidate the proximate and ultimate causes of altitudinal variations in lizard life history.

## Abbreviations

*T*_b_: body (cloacal) temperature
*T*_g_: ground surface temperature
*T*_a_: air temperature
